# Fizz-computed tomography as a novel modality of objective esophageal hiatal assessment

**DOI:** 10.1007/s00423-025-03647-2

**Published:** 2025-02-26

**Authors:** Daniel Leonard Chan, Grace Huan Yin, Manish Chug, Annemarie Hennessy, Jim Iliopoulos, Michael Leonard Talbot

**Affiliations:** 1 School of Medicine, Western Sydney University, Locked Bag 1797, Penrith, NSW 2751 Australia; 2 Department of Surgery, St George Hospital, Kogarah, NSW 2217 Australia; 3 Saint George and Sutherland Clinical School, Faculty of Medicine, The University of New South Wales, St George Hospital, Kogarah, NSW 2217 Australia; 4 Upper GI Surgery, Saint George Private Hospital, Suite 3, Level 5, 1 South Street, Kogarah, NSW 2217 Australia; 5 Rouse Hill Radiology, T5/4-6 Commercial Road, Rouse Hill, NSW 2155 Australia; 6 Faculty of Medicine and Health, The University of Sydney, Camperdown, NSW 2050 Australia

**Keywords:** Hiatal hernia, Reflux, Abdominal imaging, Diagnosis, Upper GI surgery

## Abstract

**Background:**

Traditional investigations of esophageal hiatal assessment for reflux disease and hiatal hernia (HH), such as endoscopy and barium swallow are subjective. High resolution manometry (HRM) limits hiatal hernia assessment to vertical length. We report a novel use of 3D volumetric Computed Tomography with effervescent oral contrast (Fizz-CT) as a means of preoperative HH diagnosis.

**Methods:**

A pilot series of 12 consecutive patients who underwent preoperative Fizz-CT assessment, as well as a combination of traditional investigations for HH (five primary, seven revisional HH).

**Results:**

The median age was 70years (IQR 57.5-76.8years) and median BMI 28.62 kg/m^2^ (IQR 24.9–34.1 kg/m^2^). Seven patients (58%) had a recurrent HH and five patients (42%) had a primary hiatus hernia. Fizz-CT was able to diagnose the HH in all cases. The median oesophageal hiatal surface area (HSA) was 9.46cm^2^ (IQR 4.66-13.79cm^2^). The median HH sac volume was 36.3cm^3^ (IQR 26.0-80.3cm^3^). All patients had a least one other investigation that has been traditionally used to diagnose HH. Seven of the 12 patients subsequently underwent laparoscopic HH repair surgery with intraoperative findings further confirming the radiological diagnosis of hiatus hernia.

**Conclusion:**

Fizz-CT imaging is a novel and accurate means of objective esophageal hiatal assessment in both primary and revisional HH patients. Vertical and radial measures of hiatal defects as well as hernia volumetry can be obtained. In post-surgical patients the relationship between the esophago-gastric junction and an infra- or supra-diaphragmatic fundoplication can also be assessed.

## Introduction

Hiatus hernias (HH) occur when contents of the abdominal cavity protrude into the mediastinum of the thoracic cavity through the oesophageal hiatus. Patients can suffer from significant morbidity, including gastroesophageal reflux disease (GERD), gastric ulceration respiratory complications, and gastric volvulus [1, 2]. There are four types of HH: type I– sliding, type II– paraesophageal, type III– combined types I and II, and type IV– giant HH (containing at least half of the stomach and/or another intra-abdominal organ), which each type presenting with varying symptoms and complications [3]. The severity of potential complications necessitates prompt and accurate diagnosis.

Accurate and objective preoperative investigation for HH diagnosis is important to guide patient management, and the informed consent process, however there is no current consensus on the optimal investigation. Though endoscopy can be accurate in rigorous centres, both this and barium swallow x-ray are subjective investigations [4]. Whilst the dynamic component of barium swallow x-ray offers real-time visualisation, the barium swallow protocol, interpretation, and reporting terminology are subjective and non-standardized [5]. A meta-analysis of endoscopy, barium swallow, and high-resolution esophageal manometry (HRM) suggested that the later may have better diagnostic performance [6]. However, these investigations are relatively subjective and the availability of HRM is limited to specialised centres, and poor catheter tolerance has been reported in a significant proportion of patients [7]. HRM will accurately relate the position of the lower esophageal sphincter to the diaphragmatic crura. However, elucidation of anatomy outside the esophageal lumen, such as radial separation of the crus off the esophagus, a paraesophageal hernia, or post-surgical changes, may be missed.

Computed tomography (CT) imaging for HH diagnosis has been reported for decades but has been viewed as an incidental or secondary finding rather than as a means of primary investigation for this pathology [8, 9]. Multi-planar CT with objective measurement of the double-oblique plane of the oesophageal hiatus surface area (HSA) has been reported and validated against in-vivo measurements [10, 11]. The HSA cut-off of 3.5cm^2^ demonstrated an 81% sensitivity and 88% specificity for the presence of HH in the pilot study. In a retrospective series of patients undergoing HH surgery, traditional CT reporting had a 80.4% sensitivity, and utilizing CT with HSA measurement had a 94.6% sensitivity rate [12]. Both traditional reporting and quantitative measurements had higher sensitivities than all other preoperative investigations (HRM, and gastroscopy).

Volumetric CT imaging with effervescent oral contrast (Fizz-CT) has also been recently utilized for foregut assessment, particularly for complications following bariatric surgery [13, 14]. The use of effervescent oral contrast may facilitate improved visualisation and aids 3D reconstruction of the anatomy surrounding the oesophageal hiatus. This study reports the use of novel use of Fizz-CT for objective investigation for of the oesophageal hiatus and diagnosis of HH.

## Methods

This study is a retrospective analysis of a cohort of consecutive patients from April-August 2022. All patients underwent pre-operative Fizz-CT assessment, as well as a combination of traditional HH investigations (endoscopy, barium x-ray, and HRM). Patients were identified form a prospective hiatal hernia database with data generated from an electronic medical records system. Patient demographics, preoperative investigations, and Fizz-CT findings were identified. Inclusion criteria were patients that were ≥ 18years of age, who underwent investigations for either primary or recurrent HH, for GERD or respiratory symptoms. Exclusion criteria were patients < 18years of age, declined consent to participate, or declined Fizz-CT imaging.

Fizz-CT patient preparation involves a 12-hour nil by mouth fast to ensure a completely empty stomach. Generally, a morning appointment was arranged due to fasting. The patient ingests a 30 ml mixture of a two-part carbon dioxide, gas-producing compound (X-Evess oral liquid^TM^). Part 1 is a semi-opaque white liquid that contains sodium bicarbonate 2.5 g in 500 ml. Part 2 is a blue liquid contains citric acid anhydrous 2 g in 500 ml. Alternate agents include effervescent granules (such as EZ-Gas II^®^) which has the same agents in anhydrous form swallowed with water. The oral liquid mixture should be ingested as fast as tolerated, and the patient assuming the supine and decubitus position with arms overhead as soon as possible. The scan should perform promptly and prior to any burping or regurgitation. This normally requires two radiographers (one for patient positioning, one another performing the imaging). In the supine position, imaging should be performed from above the carina down to the duodenum, this allows or detection of a HH if present.

All CT imaging was reviewed for the presence of HH as determined by the gastroesophageal junction being above the diaphragm as denoted by surrogate markers of the angle of His, change in tubular contour, visible gastric rugae. Consistent with published literature, HH was considered present if the contour abnormality was > 2 cm above the level of the diaphragm, and then subcategorized into types (I-IV) [11, 15].

Post-hoc analysis protocol - CT post-processing and analysis were performed using Sectra PACS. The oesophageal HSA (cm^2^) is calculated on a two-dimensional double-oblique image following multi-planar reconstruction (MPR), in accordance with the method described by Ou Yang et al. [11]. The HSA cut-off of 3.5cm^2^ was utilized to diagnose the presence of HH on Fizz-CT. If a HH is present, a separate volume of the hernia sac (cm^3^) was generated using Sectra PAC’s 3-D core solution and 3-D volume rendering integrated software following MPR of the images. If needed, distance between diaphragm and apparent esophago-gastric junction can be measured and the presence of any supra diaphragmatic stomach.

Statistical analyses performed using IBM Corp. Released 2020. IBM SPSS Statistics for Windows, Version 27.0. Armonk, NY: IBM Corp. Continuous variables were assessed for normality of distribution using the Shapiro-Wilk test and presented as mean ± standard deviation (range) or median (interquartile range (IQR) and categorical data as number (percentage). Comparative analysis performed by the t-test and Chi-squared test where appropriate. Intraoperative findings were the reference (“gold standard”) of HH presence or absence. A p-value of < 0.05 was considered statistically significant. The study was conducted per the Declaration of Helsinki Ethics and all patients provided their informed consent for participation [16]. The study protocol was approved by the Ramsay Health Care Human Research Ethics Committee A (approval no. 2024/ETH/0011).

## Results

A pilot series of 12 consecutive patients who underwent preoperative Fizz-CT assessment, as well as a combination of traditional investigations for HH. The median age of 70years (IQR 57.5-76.8years) and median BMI 28.62 kg/m^2^ (IQR 24.9–34.1). Seven patients (58%) had a recurrent HH and five patients (42%) had a primary hiatus hernia. Ten patients (83%) had GERD as their primary presenting symptom and the remaining two patients (17%) had respiratory and post-prandial pressure symptoms. No complications were noted from Fizz-CT investigation.

**Fig. 1:**
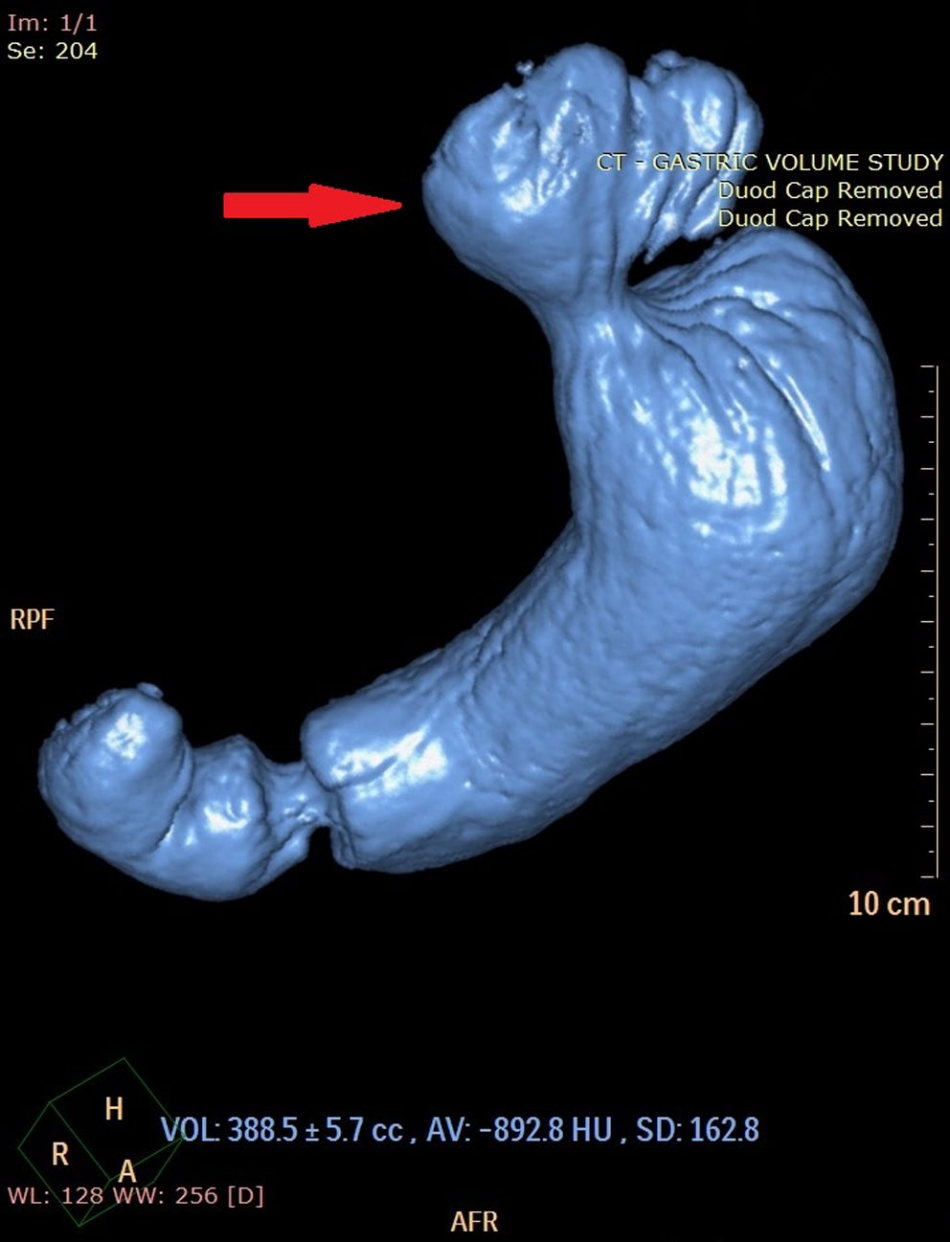
Preoperative investigation of patient #1 (76-year-old female with reflux symptoms) with a coronal image of CT (left) and 3D volumetric rendering of the same Fizz-CT (right) showing a primary hiatus hernia (red arrow)

Of these patients six (50%) had a type I (sliding) HH, five (42%) had a type II (paraesophageal) HH, one (8%) had a type IV (giant) HH. The median oesophageal HSA was 9.46cm^2^ (IQR 4.66cm^2^-13.79cm^2^). The median HH sac volume was 36.3cm^3^ (IQR 26.0cm^3^-80.3cm^3^), with one case excluded due to inability to identify the HH sac due to diffuse oesophageal dilation. Individual patient demographics and details of HH findings are summarized in Table 1. Figure 1 demonstrates the 3D reconstruction of a Fizz-CT in a 76year old female (Patient #1) with a primary HH. This patient had a HSA of 17.02cm^2^ and HH sac volume of 80.3cm^3^.

**Table 1:** Patient demographic and quantitative findings on volumetric CT with effervescent oral contrast

Patient	Age	Sex	GERD	Primary or recurrent hernia	Detected on fizz-CT	Hiatus hernia type (I-IV)	Hiatal surface area (cm2)	Hiatus hernia sac volume (cm3)
1	76	F	Y	Primary	Y	I	17.02	80.3
2	65	F	Y	Primary	Y	I	3.59	8.3
3	68	F	N	Primary	Y	IV	7.72	339
4	55	F	Y	Primary	Y	I	7.46	34.6
5	77	M	Y	Primary	Y	II	5.67	21.7
6	84	F	N	Recurrent	Y	II	4.32	†
7	65	F	Y	Recurrent	Y	I	14.23	83.1
8	72	F	Y	Recurrent	Y	II	3.61	26
9	49	F	Y	Recurrent	Y	II	11.41	33.5
10	49	F	Y	Recurrent	Y	I	18.62	51.6
11	83	F	Y	Recurrent	Y	II	11.2	36.3
12	73	F	Y	Recurrent	Y	I	12.45	60.8

† Hernial sac could not be confidently identified, diffuse oesophageal dilatation

All patients had a least one other preoperative investigation that has been traditionally used to diagnose HH demonstrating a positive finding. HRM diagnosed a HH in six of the 10 patients it was performed (sensitivity of 60%) (Table 2). The range of the vertical height of the HH was 0.4–5.4 cm. Endoscopy was performed in ten patients and diagnosed a HH in eight patients (sensitivity of 80%). Barium swallow diagnosed a HH in three patients (75% sensitivity).

**Table 2:** Detection of hiatal hernia in other investigations and confirmation with intraoperative findings

Patient	Barium swallow x-ray	Gastroscopy	High resolution esophageal manometry	Fizz-CT	Intraoperative confirmation of findings
1	-	Yes	No	Yes	Yes
2	-	No	Yes, 0.4 cm	Yes	-
3	Yes	Yes	No	Yes	Yes
4	-	Yes	-	Yes	-
5	-	No	No	Yes	Yes
6	-	Yes	Yes, 1 cm	Yes	-
7	No	-	Yes, 4.4 cm	Yes	Yes
8	-	Yes	Yes, 5.4 cm	Yes	Yes
9	Yes	-	Yes, 2.1 cm	Yes	-
10	-	Yes	No	Yes	Yes
11	Yes	Yes	Yes, 3.0 cm	Yes	Yes
12	-	Yes	-	Yes	-

Seven of the 12 patients subsequently underwent laparoscopic hiatal hernia repair surgery with intraoperative findings further confirming the radiological diagnosis of hiatus hernia. The remaining five patients are being managed with medical therapy and have not proceeded to surgery.

## Discussion

While accurate and objective preoperative investigation for HH diagnosis is important to guide patient management, the investigations currently utilized have limitations in their subjectivity, availability and tolerance. The systematic review and meta-analysis by Li et al. (2020) on the diagnostic value of current investigations for hiatal hernia conclusion is also interpreted within the limitations of only three HRM studies with 313 patients available for that analysis [6]. Another more recent study examining HRM in 67 patients demonstrated a sensitivity of 48.7% (95% CI, 31.9–65.6%), which was lower than the pooled sensitivity 95% confidence interval of the meta-analysis (0.70–0.83%) [4]. The availability of HRM is limited to specialised centres, and poor catheter tolerance has been reported in a significant proportion of patients [7].

Regardless, the quantitative measurement of HH provided by both endoscopy and HRM is the vertical distance between the separation of the gastroesophageal junction and the crural pillars of the diaphragm. Whilst it is recognized that these investigations can provide additional diagnostic information with mucosal tissue biopsy and on esophageal motility respectively, they provide little information on the esophageal hiatus and may report HH diagnosis as a secondary function of the investigation or even an incidental finding. Pathological dilation of the esophageal hiatus is the conceptual equivalent the fascial defect, in ventral hernias. Undoubtedly, a significant complicating factor in HH being that the hernia defect cannot be completely obliterated as in the case of ventral hernias in order to allow for the passage of the distal esophagus and vagus nerves.

Thin slice helical CT imaging has been increasingly used as a non-invasive investigation in the past two decades in both the elective and emergency settings [17]. Additionally, the efficacy of 3D image post-processing has been used to provide highly accurate and reproducible measures of complex anatomic structures, including the esophageal hiatus [11]. Ouyang et al. developed a novel method for in vivo measurement of the esophageal HSA in its unique double oblique anatomic plane. Their study established a HSA cut-off of greater than 3.5cm^2^ has 81% sensitivity and 88% specificity for the presence of HH. Our group published an external validation in a patient cohort that underwent laparoscopic HH repair surgery and found an even higher sensitivity (94.6%) in HH diagnosis when using the same cut-off [12]. In that previous study, the sensitivity of CT for HH detection was 80.4% for conventional CT and reporting with HSA calculations, and this is compared with sensitivities of 70.9% for barium swallow, 77.4% for endoscopy, and 75.3% for HRM [12]. Certainly, there is no foreseeable reason the accuracy of HH diagnosis would be much different from conventional CT with oral contrast if HSA calculations were included. It is emphasized that this pilot series explores the feasibility of Fizz-CT in HH detection, rather than being a comparison of diagnostic accuracy between investigations.

Effervescent oral contrast in Fizz-CT has the conceptual benefit of better visualisation of the esophageal hiatus and hernia sac through luminal distension. Certainly, this technique has been utilized in investigation of foregut pathology in esophageal cancer diagnosis, following surgical resection in gastric cancer, and increasingly commonly in bariatric metabolic surgery to assess the gastric pouch [17–19]. Our series represents a novel utilization of the Fizz-CT technique for patients presenting with GERD or respiratory and post-prandial pressure symptoms with the intent of diagnosing HH. This was achieved in both recurrent (seven patients) and primary HH (five patients) settings, with interpretation of traditional investigations being even more contentious following previous HH repair or other foregut surgical intervention [4].

As previously demonstrated (Table 2), all patients had a least one other preoperative investigation that has been traditionally used to diagnose HH demonstrating a positive finding with seven patients also subsequently having positive intraoperative confirmation. Two of these patients are discussed in further detail to discuss potential pitfalls in assessment with contradictory findings of preoperative investigations.

**Fig. 2:**
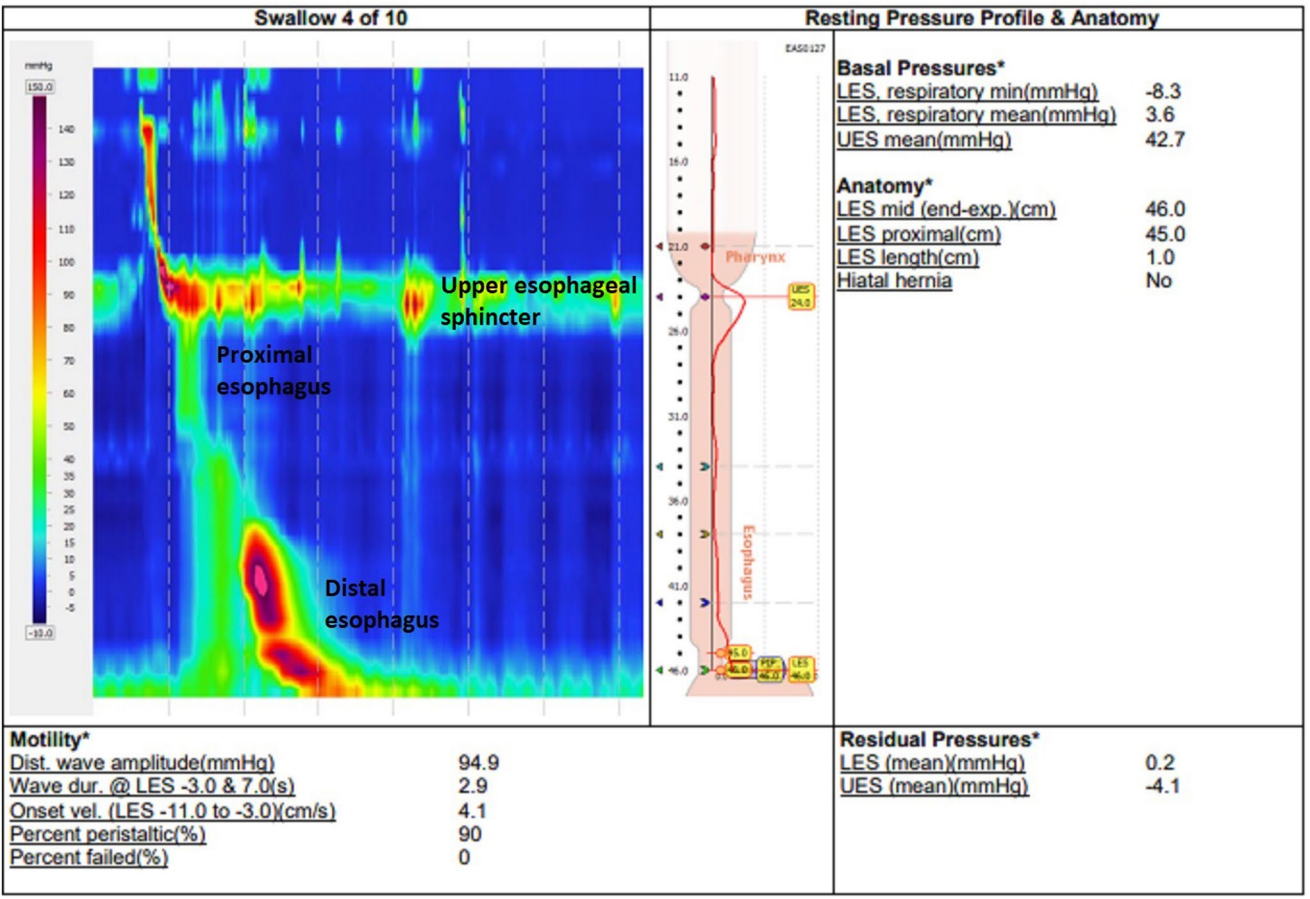
Preoperative investigation of patient #1 (76-year-old female with reflux symptoms) with the high-resolution esophageal manometry pressure topography where the manometry probe is unable to pass beyond the gastroesophageal junction and fails to demonstrate her hiatus hernia

Patient #1 is a 77year old female with GERD and a primary HH, who underwent preoperative gastroscopy, HRM, and Fizz-CT. In this patient, Fizz-CT demonstrated a type I hiatus hernia with a 17.02cm^2^ HSA and 80.3cm^3^ HH sac volume. This HH was also visible on gastroscopy. HRM did not demonstrate a HH, with the manometry probe likely bending in the HH and not truly passing below the lower esophageal sphincter (LES) and diaphragmatic crura. Figure 1 demonstrates the Fizz-CT and Fig. 2 shows the HRM in this patient. Both subsequent gastroscopy and intraoperative findings during a laparoscopic HH repair confirmed the diagnosis in this patient. This case demonstrated the limitations of HRM in assessment of larger HH, where the manometry probe may be difficult to pass below the gastroesophageal junction.

**Fig. 3:**
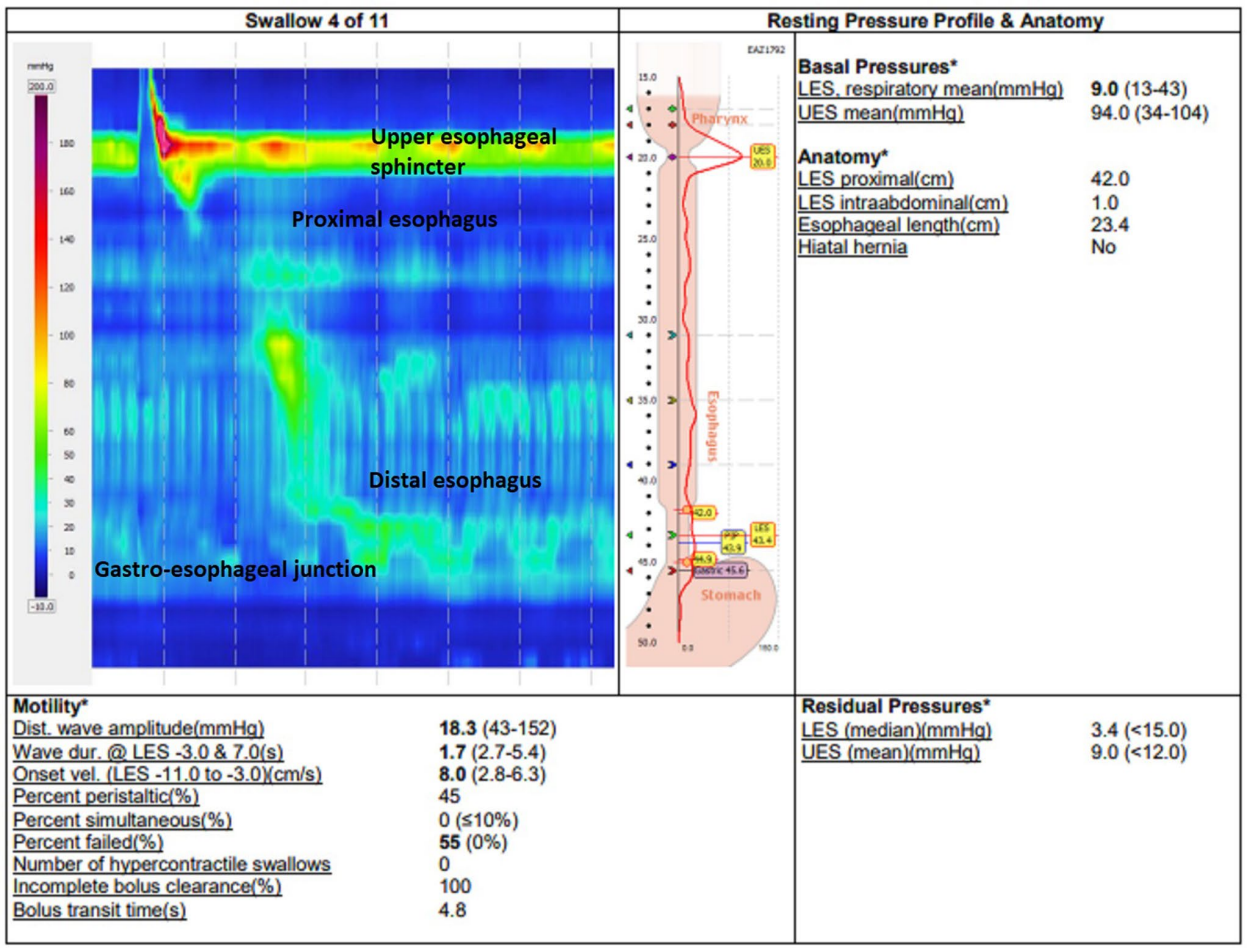
Preoperative investigation of patient #5 (77-year-old male with reflux symptoms) with high-resolution esophageal manometry pressure topography that does not show a separation of gastroesophageal junction and crural pressure zones and fails to demonstrate his hiatus hernia

**Fig. 4:**
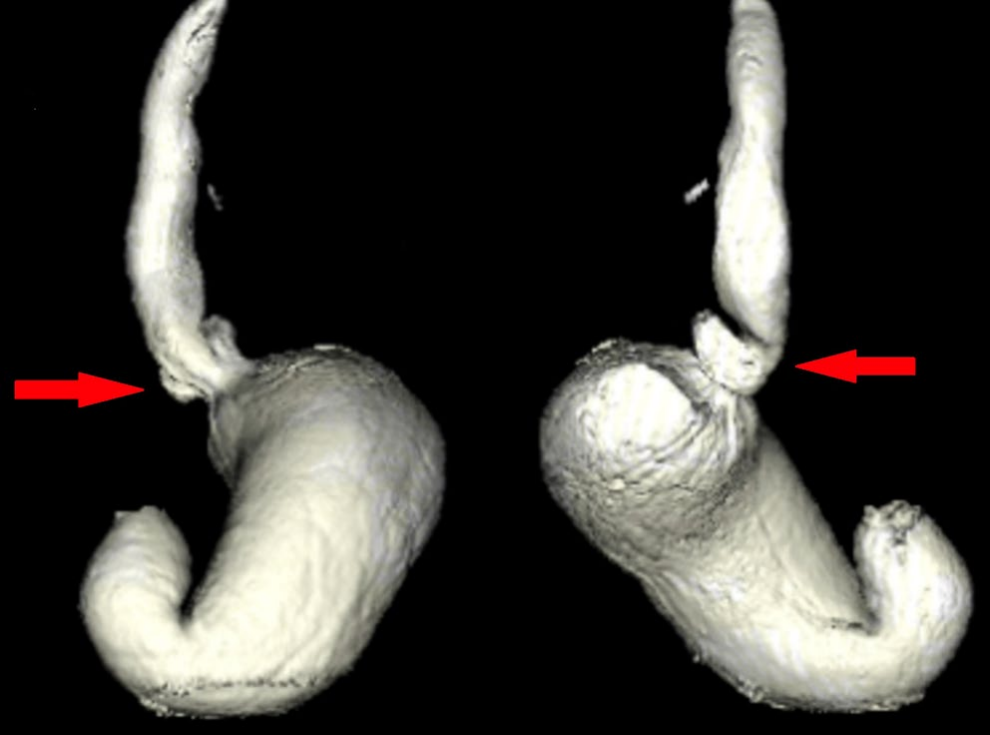
Preoperative investigation of patient #5 (77-year-old male with reflux symptoms) with 3D volumetric rendering of a Fizz-CT demonstrating a primary hiatus hernia (red arrow)

Patient #5 is a 77year old male with GERD and a primary HH, who underwent a gastroscopy, HRM and Fizz-CT as part of their preoperative assessment. Both the gastroscopy and HRM failed to demonstrate a HH in this patient. The HRM pressure topography (Fig. 3) and Fizz-CT imaging (Fig. 4) of this patient are included. The HRM pressure topography shows no clear separation of the LES and diaphragmatic crura pressure signals. However, Fizz-CT in this patient demonstrated a type II HH, with quantification showed a 5.67cm^2^ HSA and 21.7cm^3^ HH sac volume. The HH diagnosis in this patient was confirmed with intraoperative findings during a subsequent laparoscopic HH repair, following which the patient had a complete resolution of his GERD symptoms. This case demonstrated the potential limitations of gastroscopy and HRM in detecting small type II HH with paraesophageal herniation, that becomes evident in Fizz-CT.

The radiation exposure of a Fizz-CT is identical to that of conventional CT imaging of the abdomen and pelvis at approximately 16mSv, which is many times higher in comparison to the 0.32mSv of barium swallow x-ray, and radiation-free investigations of endoscopy and HRM [20, 21]. Further studies into reducing the dose of ionizing radiation to as low as reasonably achievable may include limiting the field of study to the upper abdomen and lower chest.

This pilot case series demonstrates feasibility of Fizz-CT in quantitative assessment of the esophageal hiatus and diagnosis of HH. Promisingly, the use of conventional CT to follow-up patients who have undergone HH surgery is increasingly adopted [22]. Effervescent per oral contrast provides a theoretical advantage in visualisation of this complex anatomical area, particularly in sliding (type I) HH, but whether this is of benefit over conventional CT with oral contrast is unclear. Certainly, a larger cohort of anti-reflux or bariatric surgery patients undergoing Fizz-CT, and traditional investigations and even standard CT imaging would be required to ascertain the sensitivity, specificity and accuracy of this technique. The technique may have value, not just in clinical assessment of patients undergoing foregut surgery, but also as a way to better to determine integrity of the hiatal repair in future comparative studies involving hiatal hernia surgery.

## Conclusion

This pilot series demonstrates Fizz-CT may be employed as a novel non-invasive, quantitative means of objective esophageal hiatal assessment and diagnosis of HH.

## Data Availability

No datasets were generated or analysed during the current study.
